# Impact of molecular testing on thyroid nodule neoplastic diagnosis, stratified by 4-cm size, in a surgical series

**DOI:** 10.1038/s41598-019-52581-z

**Published:** 2019-11-28

**Authors:** Rupendra T. Shrestha, Muhammed Kizilgul, Maryam Shahi, Khalid Amin, Maria R. Evasovich, Lynn A. Burmeister

**Affiliations:** 1 Department of Medicine, University of Minnesota, Minneapolis, USA; 2 Department of Surgery, University of Minnesota, Minneapolis, USA; 3 Department of Pathology, University of Minnesota, Minneapolis, USA; 4 Department of Endocrinology and Metabolism, UHS Diskapi Training and Research Hospital, Ankara, Turkey

**Keywords:** Thyroid diseases, Thyroid cancer

## Abstract

Whether molecular testing adds diagnostic value to the evaluation of thyroid nodules 4-cm or larger is unknown. The impact of molecular testing on cytopathologic-histopathologic diagnosis of neoplasm (adenoma or malignant), stratified by nodule size <or≥ 4-cm, was analyzed from a surgical series. Of 490 index nodules, molecular testing was performed on 18% of 353 nodules <4-cm and 8.8% of 137 nodules ≥4-cm (p = 0.0118). Adenoma was higher (30% vs 14%) and malignancy lower in nodules ≥4-cm vs <4-cm (p < 0.0001). Molecular testing impacted the finding of malignancy in the <4-cm group. Molecular testing of the ≥4-cm AUS and FN cytology subcategory impacted neoplasm discovery (combining adenoma and malignancy), with mutation positive 100% (3/3), mutation negative 38% (3/8), no mutation testing 88% (21/24), p = 0.0122. In conclusion, more adenoma was found in nodules ≥4-cm, including those with benign cytology, which was not explained by available molecular testing results. Molecular testing impacted the finding of malignancy in thyroid nodules <4-cm. The overall number of ≥4-cm nodules with molecular testing in this study was too low to exclude its diagnostic value in this setting. Further study is recommended to include molecular testing in nodules ≥4-cm, including those with benign cytology, to identify follicular adenoma.

## Introduction

Treatment of thyroid nodules ≥4-cm is controversial. The 2015 American Thyroid Association guidelines state it is unclear if thyroid nodules ≥4-cm and benign cytology should be managed differently than those with smaller nodules^1^.

Pathogenic driver mutations are now recognized to be important in the pathogenesis and classification of thyroid malignancy^2,3^. The use of molecular testing to guide therapeutic decision-making is evolving^4^. The impact of molecular testing on the histopathologic outcome of thyroid nodules, in relationship to nodule size, has not been described. Perhaps molecular testing of nodules ≥4-cm would lead to an increased diagnostic yield of neoplasm or malignancy in operated patients. We have previously reported a lower rate of malignancy in a surgical population of thyroid nodules ≥4-cm compared with <4-cm^5^. This study aimed to determine the impact of molecular testing, stratified by thyroid nodule size (<or ≥4-cm), on the histopathologic diagnosis of neoplasm (adenoma and malignancy) in the same surgical population.

## Methods

The study was approved by the University of Minnesota Institutional Review Board and was carried out in accordance with relevant guidelines and regulations. At the time of entry into the health system, subjects gave consent for inclusion of their data in research. The IRB does not require a repeat study-specific consent for retrospective anonymous chart review, such as was used here. Consecutive thyroidectomies performed at the university medical center, a tertiary referral hospital, between January 2010 and December 2014 were retrospectively reviewed as previously described^5^. Exclusions included age <18 years, surgery performed only for treatment of hyperthyroidism or solitary hot nodule, and nodules without well documented FNAC (100 patients). Subjects with Graves’ disease or toxic multinodular goiter but who also had discovery of thyroid nodule leading to FNAC and surgery were not excluded. Each individual is represented once, even if they had more than one thyroid operation.

All patients underwent US guided FNAC of index thyroid nodules, chosen by the treating providers. Molecular testing, when performed, was also at the discretion of the treating physician, except for a 2-year period of time during which mutation panel testing was part of a clinical pathway to automatically obtain molecular testing on indeterminate cytology as previously described^6^.

Data were recorded from preoperative thyroid US size determination and FNAC results. If a patient had more than one biopsy of the same nodule, the first FNAC, and corresponding molecular, if performed, was used in the analysis. If more than one nodule was biopsied in a given subject, the largest nodule greater than 4-cm was selected as the index nodule, or, if all nodules were under 4-cm, the nodule with most abnormal cytology was selected as the index nodule. All FNAC were classified by one of the six 2008 Bethesda categories: nondiagnostic, benign, atypia of undetermined significance (AUS)/follicular lesion of undetermined significance (FLUS), follicular neoplasm (FN)/suspicious for follicular neoplasm (SFN), suspicious for malignancy and malignant^7^. The decision for surgical removal of thyroid containing the index nodule was made at the discretion of the treating physicians, where it may have been influenced by molecular testing or other parameters. Surgical histopathology was subdivided into benign vs neoplastic (including adenoma and malignant) categories.

In the analysis, only malignancy diagnosed in the index nodule subjected to FNAC was reported as thyroid cancer for an individual subject. Incidentally discovered occult malignancy was not included since the focus of the study was to correlate cytology, histology and molecular results on the same nodule. Neoplasm analysis combined both the malignant and adenoma histopathology groups.

Patients were divided into two groups according to the sonographic size of the index nodule, ≥4-cm or under 4-cm at the time of the first FNAC. We compared FNAC results with final surgical histopathology and molecular results. Time to surgery was defined as the time interval between the index FNAC and the operation.

## Statistical analysis

Statistical analysis was performed using JMP Pro v. 13 software (SAS Institute, Cary, NC). Continuous data were reported as the median ± interquartile range (IQR) and categorical data as count and proportions.

Wilcoxon/Kruskal-Wallis test was used to compare nonparametric continuous variables. Chi square or Fisher exact test was used to compare categorical data including malignancy rates across cytologic categories by index nodule maximum diameter size <or> 4-cm or by molecular testing category. All tests were two-sided. A p-value of less than or equal to 0.05 was considered significant.

## Results

**Figure 1:**
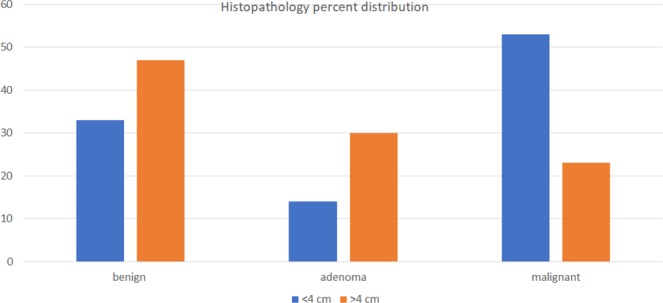
Over 4-cm nodules have higher rate of adenoma and lower rate of malignant histopathology, compared with <4-cm nodules. p < 0.0001 comparing < 4-cm to ≥ 4-cm nodules overall histopathology pattern. p < 0.0001 comparing malignancy rates 53% (188/353) < 4-cm vs 23% (31/137) ≥ 4-cm, and comparing adenoma rates 14% (49/353) < 4-cm vs 30% (41/137) ≥ 4-cm.

From a surgical series of 590 patients undergoing thyroidectomy, after excluding 100, 490 patients with preoperative FNAC were identified for analysis. The index nodule size was <4-cm in 353 (72%) and ≥4-cm in 137 (28%) of the thyroidectomy patients. On average, surgery was performed sooner after the <4-cm index nodule FNAC (median 2.2 months [IQR 1.3–4.8]) than after the ≥4-cm FNAC (median 2.9 months [IQR 1.6-8.3], p = 0.0073). Final histopathology distribution of the index nodules was 45% (219/490) malignant, 18% (90/490) adenoma and 37% (181/490) benign. Malignancy was present in 53% (188/353) of the index nodules <4-cm and in 23% (31/137) of the index nodules ≥4-cm (p < 0.0001) (Fig. 1). Benign neoplasm (i.e., adenoma) histopathology was present in 14% (49/353) of nodules <4-cm and 30% (41/137) of nodules ≥4-cm (p < 0.0001).

There was no difference in the distribution of histopathologic diagnosis by 4-cm size cut off group within each cytologic subcategory analyzed (Table 1).

**Table 1:** Distribution of surgical histopathology by cytology and size cut-off groups.

Cytologic category	Surgical histopathology n (% of cytologic size category)						p
	benign				malignant		
	benign <4-cm	benign ≥4-cm	adenoma <4-cm	adenoma ≥4-cm	<4-cm	≥4-cm	
Benign n = 145	56/68 (82%)	52/77 (68%)	8/68 (12%)	21/77 (27%)	4/68 (5.9%)	4/77 (5.2%)	0.0652
AUS/FLUS and FN/SFN n = 163	49/128 (38%)	8/35 (23%)	38/128 (30%)	17/35 (49%)	41/128 (32%)	10/35 (29%)	0.0904
Suspicious for malignancy n = 59	4/54 (7%)	0/5 (0%)	2/54 (3.7%)	0/5 (0%)	48/54 (89%)	5/5 (100%)	1.000

Benign, adenoma or malignant histopathology distribution was not different by 4 cm size cut-off across within a given cytology category.

The p statistic represents chi square Fisher’s exact test (2 tailed) comparing % histopathologic diagnosis based on index nodule size <4 vs ≥4-cm across the cytologic category row.

abbreviations: AUS = atypia of undetermined significance, FLUS = follicular lesion of undetermined significance, FN = follicular neoplasm, SNF = suspicious for follicular neoplasm.

**Figure 2:**
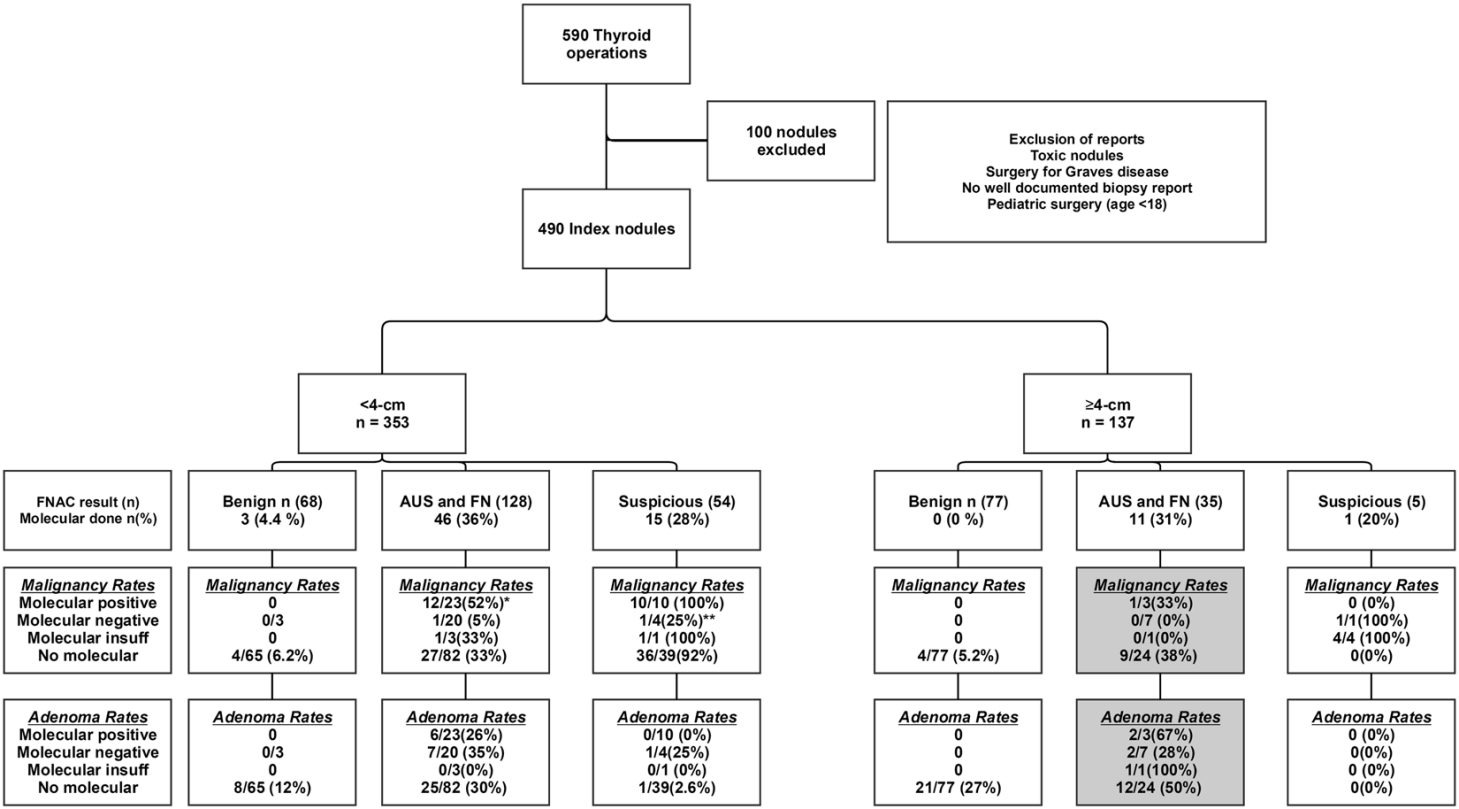
*p = 0.0042 comparing malignancy rates across the 4 molecular results groups. **p = 0.0074 comparing malignancy rates across the 4 molecular results groups. shaded p = 0.0122 comparing neoplasm rates (malignancy plus adenoma) across the 4 molecular results groups.

Molecular testing was performed on 16% of the index nodule population (76/490), including 18% (64/353) < 4-cm and 8.8% (12/137) ≥4-cm, p = 0.0118 (Fig. 2). 7 gene panel molecular test was performed in 23/76 (30%), Thyroseq. 1 in 39/76 (51%), Thyroseq. 2 in 6/76 (7.9%) and BRAF single gene mutation testing in 8/76 (11%). There was no statistical difference in the frequency of molecular testing by index nodule size <or ≥ 4-cm when analyzed across the cytologic subgroups. A mutation or fusion was identified in 47% (36/76) of those tested, including 52% (33/64) < 4-cm and 25% (3/12) ≥ 4-cm (p = 0.1634). For the whole study population, the malignancy rate was 64% (23/36) for mutation positive nodules, 8.6% (3/35) for mutation negative nodules, 40% (2/5) for molecular insufficient samples and 46% for nodules that did not have molecular testing (p < 0.0001, Table 2). This pattern of statistical significance was retained in the <4-cm group where malignancy was present in 67% (22/33) mutation positive, 7.4% (2/27) mutation negative, 50% (2/4) molecular insufficient and 56% (162/289) that did not have molecular testing (p < 0.0001). For the <4-cm AUS/FLUS and FN/SFN cytology group, positive mutation status significantly increased the yield of malignancy to 52% (12/23) compared with negative mutation 5% (1/20) or no mutation testing (33%, 27/82, p = 0.0042) (Fig. 2). For the <4-cm suspicious for malignancy cytology group, negative molecular mutation significantly reduced malignancy rate to 25% (1/4 negative mutation) compared with 100% (10/10 positive mutation) and 92% (36/39) no mutation testing, p = 0.0074) (Fig. 2).

**Table 2:** Neoplasm and malignancy rates with molecular testing.

	Rate (%)	Molecular testing not done	Molecular testing done			p
Malignancy	219/490 (45%)	191/414 (46%)	28/76 (37%)			0.1671
Neoplasm	309/490 (63%)	262/414 (63%)	47/76 (62%)			0.7976
**Malignancy**		**No molecular**	**Insufficient**	**Mutation +**	**Mutation −**	
overall	219/490 (45%)	191/414 (46%)	2/5 (40%)	23/36 (64%)	3/35 (8.6%)	<0.0001
<4-cm	188/353 (53%)	162/289 (56%)	2/4 (50%)	22/33 (67%)	2/27 (7.4%)	<0.0001
≥4-cm	31/137 (23%)	29/125 (23%)	0/1	1/3 (33%)	1/8 (13%)	0.6822
**Neoplasm**						
overall	309/490 (63%)	262/414 (63%)	3/5 (60%)	31/36 (86%)	13/35 (37%)	0.0002
<4-cm	237/353 (67%)	197/289 (68%)	2/4 (50%)	28/33 (85%)	10/27 (37%)	0.0006
≥4-cm	72/137 (53%)	54/125 (52%)	1/1 (100%)	3/3 (100%)	3/8 (38%)	0.2197
**Adenoma**						
overall	90/490 (18%)	71/414 (17%)	1/5 (20%)	8/36 (22%)	10/35 (29%)	0.2608
<4-cm	49/353 (14%)	35/289 (12%)	0/4	6/33 (18%)	8/27 (30%)	0.0728
≥4-cm	41/137 (30%)	36/125 (29%)	1/1 (100%)	2/3 (67%)	2/8 (25%)	0.2250

Neoplasm includes the benign adenoma and malignancy group.

For the ≥4-cm group, where 23% (31/137) overall were malignant, there was only one malignant NRAS mutation positive nodule≥4-cm (33% (1/3) mutation positive), and the statistical impact of molecular testing to detect malignancy (p = 0.6822), adenoma (p = 0.2250) or neoplasm (p = 0.2197) was not demonstrated (Table 2). The adenoma rate was 18% (6/33) for mutation positive nodules <4-cm and 67% (2/3) for nodules ≥4-cm (p = 0.1176). The two mutation-positive ≥4-cm adenomas had fusion PAX8/PPARG and mutant HRAS. In contrast to the whole ≥4-cm group, the impact of molecular testing for the ≥4-cm AUS/FLUS and FN/SFN cytology subcategory could be seen for neoplasm (combining adenoma and malignancy), with mutation positive 100% (3/3), mutation negative 38% (3/8), no mutation testing 88% (21/24), p = 0.0122 (Fig. 2). Molecular testing was not performed on ≥4-cm nodules with benign cytology.

## Discussion

In this retrospective surgical series of 490 consecutive thyroidectomies over a 5-year period comparing index nodules smaller or larger than 4-cm in size, more nodules ≥4-cm were benign and adenomatous than malignant, compared to the distribution in the <4-cm group. Importantly, adenoma was found at higher rate in the ≥4-cm nodule group (30% vs 14%) than in the <4-cm group. Likewise, in the ≥4-cm group with benign cytology the same pattern was observed, with 27% adenoma in the ≥4-cm group vs 12% in the <4-cm group. Molecular testing was associated with increased neoplastic yield (either malignancy or adenoma) only in the ≥4-cm nodules with AUS/FLUS and FN/SFN cytology. It was not associated with the surgical decision resulting in adenoma diagnosis in the setting of benign cytology nor did it increase the yield of operative malignancy in the ≥4 cm nodule group. More molecular testing was used in the <4-cm group, where it significantly increased the malignancy yield in the AUS/FLUS plus FN/SFN and where negative molecular result decreased the malignancy yield in the suspicious for malignancy cytology group.

Only 4 other series have reported on the adenoma rate of operated thyroid nodules ≥4 cm^8–11^. The overall surgical prevalence of adenoma varied widely at 6.3%^11^, 11%^10^ and 28%^9^. The current study had the highest rate of adenoma reported to date, at 30% of operated nodules ≥4-cm. For benign cytology nodules ≥4-cm, adenoma was found in 27%. Two studies reported similarly high adenoma rates (27%^8^ and 42%^9^) in ≥4-cm nodules with benign cytology. The higher rate of adenoma in the ≥4-cm group was not explained by clinical parameters or by available molecular results. Since toxic nodules were excluded based on the study inclusion criteria the study should have favored against the finding of adenoma making the high rate of adenoma found in ≥4-cm nodules more remarkable.

Molecular testing was performed on only 8.8% of the nodules ≥4-cm. In the ≥4-cm AUS/FLUS and FN/SFN group, molecular testing increased the yield of finding neoplasm (combined adenoma and malignancy) but not either histopathologic diagnosis alone. Molecular testing was not performed on any of the benign cytology nodules ultimately read as adenoma.

Molecular testing was performed in nearly twice as many nodules under 4-cm than ≥4-cm (18% vs 8.8%, p = 0.0118), an overall small fraction of the indeterminate samples. 89% of the molecular tests used a mutation panel while 11% were single gene BRAF mutations tests. The impact of molecular testing was seen in the <4-cm group as a whole as well as in subgroup analysis including the AUS/FLUS plus FN/SFN cytology group where mutation significantly increased the yield of malignancy over mutation negative and untested nodules (Fig. 2). In the <4-cm suspicious for malignancy cytology group a negative molecular result significantly reduced the risk of malignancy. Therefore, the molecular testing may have further enriched the malignancy rate in the <4-cm group while at the same time the ≥4-cm group had more surgery despite having benign cytology.

The shorter time to surgery for smaller nodules may reflect the impact of the molecular testing in the <4-cm population.

Only one other study reported molecular testing in nodules ≥4 cm^12^. In that study mutation was positive in 9/107 (9.3%) of nodules ≥4-cm, with all resulting in papillary thyroid carcinoma diagnosis.

Should thyroid nodules ≥4-cm be excised, regardless of cytology? Current meta-analysis and other studies suggest that cytology can be useful to exclude malignancy in nodules ≥4-cm, that cancer rates are not higher in nodules ≥4-cm compared with <4-cm^5,13^. However, perhaps nodules ≥4-cm should be considered for removal based on the higher rate of adenoma and the possibility that the adenoma represents a premalignant condition.

Histopathology remains the gold standard for designating a thyroid nodule as benign or neoplastic, including adenoma or carcinoma. Histopathologic interpretation is not straightforward, especially for follicular lesions of the thyroid, where even experts may disagree^14–16^.

Likewise, the concept exists of follicular adenoma as a premalignant lesion in the multi-step model of thyroid cancer tumorigenesis^17–20^. A continuous evolution from follicular adenoma to carcinoma, even if the next step transformation rate to malignancy is low, may increase the importance of surgical removal of follicular adenoma as a means to prevent thyroid cancer, analogous to removal of colon polyps as a means to prevent colon cancer.

This study has some limitations. First, it is a single center retrospective surgical series which included analysis of only those index nodules selected for surgery, not all nodules. There was a relatively small percentage of molecular testing use overall, especially in the ≥4-cm group, where the impact of molecular testing was less apparent. Factors beyond what we analyzed may have gone into the decision making for surgery. We cannot exclude selection bias to send larger benign nodules to surgery, increasing the benign denominator for this group. Still, we believe this surgical population is comparable to others previously reported. Finally, pathologists were not blinded to the results of the molecular testing and this may have influenced their histopathologic diagnosis.

In conclusion, a higher rate of adenoma was found in a surgical series of nodules ≥4-cm, including those with benign cytology, which was not explained by available molecular testing results. Molecular testing impacted the finding of malignancy in thyroid nodules <4-cm. The overall number of ≥4-cm nodules with molecular testing in this study was too low to exclude its diagnostic value in this setting. Further study is recommended to include molecular testing in nodules ≥4-cm, including with benign cytology, to explore preoperative criteria for identifying follicular adenoma.
